# Usefulness of a fast track list for anxious patients in a upper GI endoscopy

**DOI:** 10.1186/1471-2482-12-S1-S11

**Published:** 2012-11-15

**Authors:** Fabrizio Cardin, Alessandra Andreotti, Manuel Zorzi, Claudio Terranova, Bruno Martella, Bruno Amato, Carmelo Militello

**Affiliations:** 1 Department of Surgical and Gastroenterological Sciences, University of Padua, Italy; 2 Explora snc di Vittadello Fabio & C. - Ricerca & Analisi statistica Padova, Italy; 3 Istituto Oncologico Veneto, IRCCS, Padova, Italy; 4 Department of Molecular Medicine, University of Padua, Italy; 5 University of Naples Federico II - Department of General Surgery, Italy

## Abstract

**Background:**

To determine whether patients with no alarm signs who ask the endoscopist to shorten their waiting time due to test result anxiety, represent a risk category for a major organic pathology.

**Methods:**

At our open-access endoscopy service, we set up an expedite list for six months for outpatients who complained that the waiting time for gastroscopy was too long. Over this period we studied 373 gastroscopy patients. In addition to personal details, we collected information on the presence of Hp infection and compliance with dyspepsia guideline indications for gastroscopy.

**Results:**

Average waiting time was 38.2 days (SD 12.7). The 66 patients who considered the waiting time too long underwent gastroscopy within 15 days. We made 5 diagnoses of esophageal and gastric tumour and gastric ulcer (7.6%) among the expedite list patients and 14 (4.6%) among those on the normal list (p=0.31). On including duodenal peptic disease in the analysis, the total prevalence rate rose to 19.7% in the short-wait group and to 10.4% (p=0.036) in the longer-wait group.

**Discussion and conclusions:**

Our data suggests that asking to be fast-tracked does not have prognostic impact on the diagnosis of a major (gastric ulcer and cancer) pathology.

## Background

There is a well-known close association between endoscopy waiting times and test results in urgent referrals, particularly in the case of digestive bleeding [1]. Clinical progress of malignancy is also influenced by time to exam [2] and this event may have also medical-legal consequences [3]. From an organizational stand point, this evidence has led to the creation of on-call services for urgent endoscopy and implementation of the two-week rule for endoscopyreferrals.

No such association between waiting time and endoscopy outcomes has instead been observed for dyspepsia, yet endoscopists working in very busy services are often asked to fast-track patients withnon-medically urgent conditions and dyspeptic symptoms, leading to organizational problems.

Some requests are made by general practitioners but everyday experience and experimental evidence have shown that considerable pressure on specialist services is brought to bear directly by patients, particularly those affected by functional pathology [4]. The key to patient management in dyspepsia guidelines is endoscopy timing: referral is immediate in the presence of red flags but postponed until outcome of symptomatic treatment in young patients [5]. It is not clear, however,whether patient requests to be fast-tracked have prognostic value in relation to symptom severity.

Prognostic characteristics extrapolated from patient self-rating questionnaires have permitted to differentiate between the probability of a functional or organic pathology being present [6]. This observation could be further explored to assess the value of patient insistence or fear of late diagnosis. While generalized anxiety disorder is associated with functional dyspepsia [7], Hospital Anxiety Depression scale results have shown that patients affected by organic and functional pathology have comparable levels of anxiety and depression prior to endoscopy [8].

Sonnenberg studied the main principles of queueing theory to prevent under use of endoscopic resources [9] and various strategies have been studied to give priority to exams with a greater probability of yielding an organic pathology [10-12]. However, endoscopists are faced with a burgeoning array of difficult choices in managing waiting lists, particularly when patients directly ask them to shorten waiting time after bypassing the central appointment service.

The main aim of this study was to examine how accepting patient requests to reduce the waiting time set by the central appointment service impacted gastroscopy outcomes. Specifically we sought to assess whether fast-tracking patients on their request improved identification of organicpathology, to show whether insistence on rapid admission to gastroscopy could be considered an alarm sign for organic dyspepsia and, possibly, to suggest an effective management approach to these patients. Accordingly, over a period of six months, our open-access endoscopy clinic agreed to fast-track outpatients who believed their symptoms demanded prioritized medical attention.

## Methods

Over a six-month period, at an open-access service operating in Padova, an industrial town in North-east Italy with 390,000 residents, we prospectively studied the results of esophagogastro-duodenoscopy(EGDS) procedures of outpatients affected by dyspepsia, in relation to their waitingtime.

We stratified the study patients by sex, age (≤45 and >45 years), presence of Helicobacter pylori(Hp) infection and appropriateness of endoscopy referral according to international dyspepsia guidelines [13].

For each patient we calculated the time elapsing between GP referral for gastroscopy and the procedure date, classifying waiting times as ≤15 and >15 days. The study also included patient referrals for follow-up endoscopy. We differentiated waiting times by activating an expedite examination list composed of patients who, directly or through a third person, expressed concern tothe endoscopist about the length of waiting time set by the central appointment service, and askedfor prioritized medical attention.

The study excluded inpatients or patients with medically urgent conditions, as digestive bleeding, or patients referred by a gastroenterologist. To account for differences in clinical management of gastric and duodenal ulcer, we performed two sets of analysis. One included only diagnoses ofgastric ulcer and gastric or esophageal cancer. The other set also included the presence of duodenalulcer.

### Statistical analysis

The chi square (χ^2^) test was used to compare differences between the two groups and the two sets of positive gastroscopies (first set: carcinoma plus gastric ulcer; second set:carcinoma plus gastric and duodenal ulcer). If the chi squared test was not applicable, the analogous non-parametric test (Fisher’s exact test) was used. The SAS statistical software, rel. 9.1.3, was used for the analysis. A p-value<0.05 was considered significant.

## Results

According to the admission criteria, 373 gastroscopies were performed over the study period in 226 men (mean age 53.6 years, SD 15.8) and 147 women (mean age 54.2 years, SD 15.8). Table 1 shows the distribution of the diagnoses. We observed a high prevalence (3%) of malignancies(n=11). Overall, 5.1% of subjects were positive for gastric ulcer or tumour, and 26 patients had aduodenal ulcer.

**Table 1:** Distribution of diagnosed lesions (n=373)

Diagnosis	N	%
Normal	286	76.7

GERD*	40	10.7

Duodenal Ulcer	26	7.0

Gastric carcinoma†	11	3.0

Gastric Ulcer	8	2.1

Others‡	2	0.5

* Gastro-Esophageal Reflux Disease, including oesophagitis, jatal hernia

† of which 3 lymphomas

‡ 2 polyps, 1 operated stomach

Mean waiting time on the standard list was 38.2 days (SD 12.7). Sixty-six patients were placed on the expedite list.

Table 2 shows distribution of the two groups of patients by sex, age, presence of Hp infection and compliance with the dyspepsia guideline on indications for endoscopy. The only difference between the two groups was compliance with guideline indications, with expedite-list patients showing greater compliance (72.7% vs 56% p=0.012).

**Table 2:** Distribution of main variables according to waiting time.

	Subjects		Waiting time (%)		
	**N**	**%**	**≤ 15 days**	**> 15 days**	**p-value**

**Sex**					
Males	226	60.6	53.0	62.2	0.17
Females	147	39.4	47.0	37.8	

**Age (years)**					
≤ 45	103	27.6	28.8	27.4	0.81
> 45	270	72.4	71.2	72.6	

**Helicobacter Pylori**					
Negative	215	59.1	57.8	59.3	0.82
Positive	149	40.9	42.2	40.7	

**Compliance with dyspepsia guidelines**					
Yes	220	59.0	72.7	56.0	0.012
No	153	41.0	27.3	44.0	

The group of patients with a shorter wait had a higher percentage of major diagnoses, but with statistical significance in the second analysis set only (malignancies, gastric and duodenal ulcers19.7% vs 10.4% p=0.036). Differences were not statistically significant in the first analysis set,which excluded duodenal pathology (7.6% vs 4.6% p= 0.35) (Table 3)

**Table 3:** Pathologies diagnosed according to two different waiting times

	Subjects		Lesions (%)			
	**N**	**%**	**Cancer and gastric ulcer**	**p-value**	**Cancer and gastric / duodenal ulcer**	**p-value**

**Waiting time (days)**						

≤ 15	66	17.7	7.6	0.35	19.7	0.036
> 15	307	82.3	4.6		10.4	

**Figure 1:**
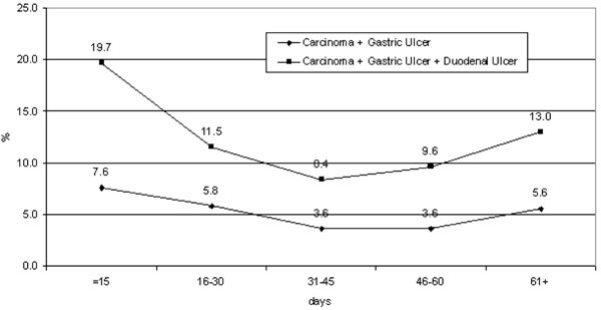
Proportion of lesions detected by waiting time.

Figure 1 shows the percentage of lesions according to waiting time, revealing a slight increase inpositive diagnoses in patients with longer waiting times.

## Discussion

Our study was prospectively designed to investigate whether patients requesting fast track gastroscopy had a higher incidence of organic pathology. We adopted broad inclusion criteria for expedite list placements in order to emphasize patient concern rather than medical priority criteria.

Our observations suggest that accepting patients’ requests to reduce waiting time, by creating aspecial expedite list to manage gastroscopies within an open-access upper-endoscopy service, doesnot provide substantial diagnostic gains.

While the two study groups were comparable in terms of recognized prognostic elements as age, Hpinfection and sex, they differed in compliance with guideline indications for endoscopy. Despite this lack of homogeneity favouring the expedite group, there was not a substantial increase in the number of diagnosed organic pathologies among the expedite list patients. Only when assessment of the positive diagnoses included duodenal ulcer was there a significant difference between the two groups in the number of organic pathology diagnoses. This may be because the study has a type-2 limit related to the small number of organic diagnoses or, as recently demonstrated, be due to a difference in the acuteness of dyspeptic symptoms for duodenal ulcer [14], or a higher prevalence of painful symptoms [15].

In any event, our case series was characterised by a higher prevalence of organic pathologies than reported in other studies [16,17]. Moreover, to avoid a type-2 error we would have had to collect an almost unrepeatable number of malignancy diagnoses. It is, however, worth stressing that the painsymptom caused by a probable duodenal ulcer is what forces patients to ask for a reduction inwaiting time [18]. In our opinion, this is an important management finding, insofar as an antacidtherapy could be prescribed to patients who cannot be fast-tracked.

We also believe our data generally confirm observations that the presence of alexithymia is associated with more severe symptoms during the week preceding endoscopy [19], although it does not differ in patients with organic and functional pathology.

Our decision to assess the importance of patient intervention in priority setting was partly influenced by explicit reference, in the latest revision of the NICE guidelines, to patients’ fear that amalignancy was present [20]. However, unlike the results of another Italian case series, reduced waiting times did not correspond to a higher number of positive diagnoses. Findings also differed because Parente’s strategy was based on participation in a regional scheme to reduce endoscopy waiting times and thus prioritization was more dependent on physician evaluation [21]. In any event, prioritizing endoscopy waiting lists in order to identify gastrointestinal malignancies earlierand in higher numbers has been criticized, even in the case of the two-week rule, because it canseriously affect endoscopy service activities by lengthening other waiting lists [22].

Conversely, our findings also showed that patients who agreed to wait longer for the procedure did not have a zero risk of malignancy. The diagnostic curves for waiting times exhibited a relative increase in positive malignancy diagnoses in patients waiting longer. This may be associated, on the one hand, with greater diagnostic yield for advanced lesions and, on the other, with the role of follow-up procedures that are booked irrespective of the presence of symptoms for the precancerous pathologies included in our study.

It would have been interesting to extend our observations to determine whether a “target wait”principle, similar to the one adopted by Smith [23], reduced the number of failures to attendendoscopy, which is another organizational problem facing endoscopy services [24]. Unfortunately,we were unable to do so. It is also difficult to weigh up the potential benefits for general healthspending that result from reducing the amount of time patients are exposed to test result anxiety.

Considering therefore that the marked increase in endoscopy workload did not lead to earlier detection of esophageal or gastric malignancy [25], in order to aid diagnostic yield and reduce time to endoscopy, it would be helpful to identify any other potential alarm symptoms that have not yet been studied but have shown poor diagnostic yield in identifying organic pathology [26,27], as weight loss, irrepressible vomiting, and age.

We relied solely on univariate analysis to derive the poor prognostic impact of patient fear of a latediagnosis. In multivariate analysis the impact of this variable would not have been independent ofother robust predictive elements, as age, sex and Hp infection.

Our findings also suggested that one method of managing patients, who ask to be fast tracked when faced with a long wait for endoscopy, is to prescribe antisecretory therapy to control pain, since theliterature suggests that this does not affect endoscopic diagnosis [28]. Alternatively, patients couldbe screened for psychiatric disorders via a structured psychiatric interview [29].

## Conclusions

While shortening waiting times in anxious patients may be effective in cutting costs, asshown by other studies [29], it does not help to identify cases of organic dyspepsia.

## Competing interest

The authors declare that they have no competing interest.

## Authors’ contributions

FC designed the study and gave a fundamental contribution to the recruitment of patients. He contributed to the literature review, to the writing and reviewing of the paper. MZ performed statistical analysis. AA and CT contributed to the analysis and interpretation of the data, to the writing and reviewing of the paper. BM, BA and CM were involved in drafting the manuscript. All the authors read and approved the final manuscript.
